# Prognostic impact of muscle mass loss in elderly patients with oesophageal cancer receiving neoadjuvant chemoradiation therapy

**DOI:** 10.1002/jcsm.13462

**Published:** 2024-04-13

**Authors:** Jeong Yun Jang, Dongryul Oh, Jae Myoung Noh, Jong‐Mu Sun, Hong Kwan Kim, Young Mog Shim

**Affiliations:** ^1^ Department of Radiation Oncology, Konkuk University Medical Center Konkuk University School of Medicine Seoul Korea; ^2^ Department of Radiation Oncology, Samsung Medical Center Sungkyunkwan University School of Medicine Seoul Korea; ^3^ Department of Medicine, Division of Hematology‐Oncology, Samsung Medical Center Sungkyunkwan University School of Medicine Seoul Korea; ^4^ Department of Thoracic and Cardiovascular Surgery, Samsung Medical Center Sungkyunkwan University School of Medicine Seoul Korea

**Keywords:** Elderly, Low muscle mass, Neoadjuvant chemoradiotherapy, Oesophageal cancer, Skeletal muscle loss

## Abstract

**Background:**

We aimed to identify the impact of muscle mass on locally advanced oesophageal cancer (LAEC) in elderly patients receiving neoadjuvant chemoradiation therapy (NACRT).

**Methods:**

We reviewed the medical records of 345 patients diagnosed with LAEC who underwent NACRT and surgery. Physical variables, including height, weight, skeletal muscle mass, and laboratory values, were obtained before and after NACRT. Body mass index (BMI, kg/m^2^), neutrophil‐to‐lymphocyte ratio (NLR), platelet‐to‐lymphocyte ratio (PLR), and prognostic nutritional index (PNI) were calculated as height/(weight)^2^, ANC/ALC, platelet count/ALC, and (10 × albumin + 0.05 × ALC), respectively. The cutoff for low muscle mass was 43.0 cm^2^/m^2^ for BMI below 25 kg/m^2^ and 53.0 cm^2^/m^2^ for BMI 25 kg/m^2^ or higher. The skeletal muscle index (SMI) was defined as skeletal muscle area/(height)^2^ (cm^2^/m^2^). The ΔSMI (%/50 days) was defined as (SMI after NACRT − SMI before NACRT)/interval (days) × 50 (days) to compare changes over the same period. The excessive muscle loss (EML) group was defined as patients with ΔSMI ≤−10% following NACRT. An elderly patient was defined as aged ≥65 years. The primary outcome measure was overall survival (OS).

**Results:**

During a median follow‐up of 32.8 months (range, 2.0–176.2), 192 patients died, with a median OS of 50.2 months. Elderly patients did not show inferior OS (young vs. elderly, 57.7% vs. 54.0% at 3 years, *P* = 0.247). 71.0% and 87.2% of all patients had low muscle mass before and after NACRT, respectively, which was not associated with OS (*P* = 0.270 and *P* = 0.509, respectively). Inflammatory (NLR and PLR) and nutritional index (PNI) values or their changes did not correlate with OS. However, the EML group had worse OS (41.6% vs. 63.2% at 3 years, *P* < 0.0001). In the multivariate analysis, EML was also a significant prognostic factor for OS. In the subgroup analysis by age, EML was a strong prognostic factor for OS in the elderly group. The 3‐year OS was 36.8% in the EML group and 64.9% in the non‐EML group (*P* < 0.0001) in elderly patients, and 47.4% and 62.1% (*P* = 0.063) in the young patients. In multivariate analysis of each subgroup, EML remained prognostic only in the elderly group (*P* = 0.008).

**Conclusions:**

EML may be strongly associated with a deteriorated OS in elderly patients undergoing NACRT, followed by surgery for LAEC. The strategies for decreasing muscle loss in these patients should be investigated.

## Introduction

Oesophageal cancer (EC) is the seventh most common and sixth most lethal cancer worldwide, with the highest incidence in individuals in their 60s and 70s, and approximately 30% of newly diagnosed patients are over 70 years old.^1^, ^2^ With an aging world population, the proportion of elderly patients with cancer has increased.^3^

Locally advanced oesophageal cancer (LAEC), in which the 5‐year survival rate rarely exceeded 40%, achieved a median overall survival (OS) of approximately 49 months with neoadjuvant concurrent chemoradiation therapy (NACRT) and surgery, owing to the success of the CROSS trial, which has been accepted as the standard for care.^4^ However, it is challenging for the elderly to receive both intensive chemoradiation and surgery. According to a population‐based study, among patients with potentially curable EC, younger ones tend to undergo NACRT and surgery. In contrast, older patients receive radiation alone or are only under observation.^5^ Elderly patients may experience postoperative complications and have a low survival rate due to underlying characteristics such as cardiopulmonary comorbidities or poor performance status.^6^ However, based on various research findings, studies suggest that old age may yield similar survival outcomes to young age, thereby remaining a controversial topic.^7^, ^8^, ^9^, ^10^ Among the baseline characteristics related to survival, sarcopenia, which represents the loss of the amount and function of skeletal muscle, has emerged as an important factor in an aging era.^11^, ^12^ In general, sarcopenia commonly occurs at an advanced age, and it can occur more frequently in patients with EC due to reduced food intake and activity during treatment or the disease itself.^13^, ^14^ Although the importance of muscle mass maintenance in patients receiving active cancer treatment has been clarified, an analysis of its impact by age group is lacking.^15^ Therefore, this study aimed to investigate the prognostic impact of muscle mass according to age group and the prognosis of elderly patients who underwent NACRT.

## Methods

### Patients

We retrospectively reviewed the medical records of patients with LAEC who underwent NACRT at Samsung Medical Center between April 2005 and April 2021. Among 805 patients, those who received the conventional fractionated radiation therapy and 5‐fluorouracil (5‐FU)/cisplatin (FP) as chemotherapeutic agents were included in the homogenous treatment group. Other key inclusion criteria were as follows: (1) patients who completed NACRT and curative surgery; (2) patients with positron emission tomography (PET) images before and after NCART; (3) male patients ≥18 years; as in the previous study (only male patients were selected due to the limited number of female patients and to ensure physiological equivalence).^16^, ^17^ Finally, data from 345 patients were analysed (Figure S1). Stage evaluation was performed according to AJCC 8th edition.

### Treatments

Computed tomography (CT) simulation images were obtained with 2.5 mm slices; most patients performed it with free‐breathing, and patients expected to have large tumour movements due to tumours located in the lower oesophagus underwent four‐dimensional CT (4D‐CT) based on the physician's decision. The gross tumour volume (GTV) was delineated, including all primary and regional lymph nodes (LN) suspected of malignancy, by referring to endoscopy, chest CT, and PET‐CT scans. The clinical target volume (CTV) for the primary tumour was delineated with margins of 5 mm radially and 2–3 cm longitudinally from the GTV and 1 cm in all directions for regional LN, and no elective nodal irradiation was performed. For patients who underwent 4D‐CT, the internal target volume (ITV) was set considering tumour movement so that the CTV could be covered in all breathing phases, and the planning target volume was delineated with a 5–7 mm expansion from the CTV or ITV. Planning was performed using 4–10 MV energy with three‐dimensional conformal radiation therapy (3D‐CRT) or intensity‐modulated radiation therapy (IMRT). Radiotherapy was administered 5 days per week, with a total of 44.0 Gy delivered at 2 Gy per fraction for 5 weeks. Image guidance was performed by weekly cone beam CT image matching.

In the FP regimen, patients received cisplatin 60 mg/m^2^/day on the first day and 5‐FU 1000 mg/m^2^/day for four consecutive days every 3 weeks. After the completion of NACRT, re‐evaluation was performed using endoscopy, chest CT, and PET‐CT scan to confirm the absence of unresectable or metastatic disease. Surgery was performed after a median of 5.0 weeks (interquartile range, 4.3–6.0) from the end of NACRT, and 2‐ or 3‐field LN dissection was completed depending on the location of the tumour.

### Data collection and outcomes

Physical parameters, including height, weight, and skeletal muscle mass were obtained. Laboratory data were collected for white blood cell count (WBC, /μL), platelet count (/μL), absolute neutrophil count (ANC, /μL), absolute lymphocyte count (ALC, /μL), and albumin (g/dL) levels using blood tests. The pretreatment value was measured at the time closest to the start of radiation treatment, and the post‐treatment value was determined as the value measured at the earliest time among those measured within 3 months after the end of NACRT. Body mass index (BMI, kg/m^2^), neutrophil‐to‐lymphocyte ratio (NLR), platelet‐to‐lymphocyte ratio (PLR), and prognostic nutritional index (PNI) were defined as height/(weight)^2^, ANC/ALC, platelet count/ALC, and (10 × albumin + 0.05 × ALC), respectively.

Data on skeletal muscle area were obtained from cross‐sectional images of the third lumbar vertebra level using PET‐CT and MATLAB version R2014a (Mathworks Inc., Natick, MA, USA) (*Figure* *S2*). The skeletal muscle index (SMI) was defined as skeletal muscle area/(height)^2^ (cm^2^/m^2^), and cutoff value for low muscle mass was determined based on Martin's criteria, with different thresholds applied according to BMI.^18^, ^19^ For patients with a BMI of <25 kg/m^2^, the cutoff was set at 43.0 cm^2^/m^2^, while for patients with a BMI of 25 kg/m^2^ or greater, the cutoff was established at 53.0 cm^2^/m^2^. Because the interval of PET‐CT performed before and after NACRT was different for each patient, the ΔSMI (%/50 days) was defined as (SMI after NACRT – SMI before NACRT)/interval (days) × 50 (days) to compare changes over the same period. The cut‐off value for excessive muscle loss (EML) and non‐excessive muscle loss (non‐EML) were defined as patients with ΔSMI of ≤−10 and >−10%, respectively, based on our previous study.^16^

The primary outcome was OS, defined as the time between the first day of NACRT and death from any cause. The secondary outcome was progression‐free survival (PFS), defined as the time between the first day of NACRT and any disease recurrence/progression or death.

### Statistical analyses

Statistical analyses were performed using R version 4.2.2 (R Development Core Team, Vienna, Austria). The Kaplan–Meier method was used to calculate the PFS and OS. Univariate and multivariate Cox regression analyses were performed to examine the risk factors influencing OS. A chi‐square test was performed for nominal variables and an independent simple *t*‐test was performed for continuous variables to compare the distribution of patient characteristics in each group according to age and muscle mass loss. Statistical significance was set at *P* < 0.05.

## Results

### Patient's characteristics

A total of 345 patients were included in the analysis, and their characteristics are shown in Table S1. All patients were male with a median age of 63 years (range, 36–83). The European Cooperative Oncology Group performance status (ECOG PS) was 0–1 in 339 patients (98.3%). There were 254 (73.6%) and 139 (40.3%) patients with clinical stages T3–4 and N2–3, respectively, and T and N‐positives were 181(52.5%) and 177 (51.3%), correspondingly. Ninety‐six patients (27.8%) had a pathologic complete response after NACRT (ypCR), and 323 patients (93.6%) underwent R0 resection. Before radiation therapy (RT), 245 patients (71.0%) had low muscle mass, which increased to 301 (87.2%) after treatment.

Table 1 shows the changes in the physical indices and laboratory values before and after NACRT. All parameters, except NLR and PLR, decreased after treatment. The mean ΔSMI for all patients was −7.0 cm^2^/m^2^ [standard deviation (SD), 6.5].

**Table 1:** Physical and laboratory measurements before and after neoadjuvant chemoradiation therapy

	Mean ± standard deviation		
	Before NACRT	After NACRT	Delta [After ‐ Before]
BMI, kg/m^2^	22.9 ± 2.8	22.0 ± 2.9	−0.9 ± 1.4
SMI, cm^2^/m^2^	47.8 ± 8.1	43.2 ± 8.1	−7.0 ± 6.5^a^
WBC, ×10^3^/μL	7.8 ± 2.3	4.7 ± 2.8	−3.1 ± 3.5
Platelet count, ×10^3^/μL	244.0 ± 69.0	186.5 ± 65.1	−57.5 ± 67.8
ANC, ×10^3^/μL	4.9 ± 2.1	3.0 ± 2.6	−1.9 ± 3.3
ALC, ×10^3^/μL	2.1 ± 0.6	1.1 ± 0.7	−1.0 ± 0.9
Albumin, g/dL	4.3 ± 0.4	3.8 ± 0.4	−0.5 ± 0.5
NLR	2.6 ± 1.6	5.7 ± 11.7	3.2 ± 11.6
PLR	125.6 ± 52.7	300.8 ± 380.3	175.3 ± 379.3
PNI^b^	53.3 ± 5.3	43.5 ± 5.5	−9.9 ± 6.5

ALC, absolute lymphocyte count; ANC, absolute neutrophil count; BMI, body mass index; NACRT, neoadjuvant chemoradiotherapy; NLR, neutrophil‐to‐lymphocyte ratio; PLR, platelet‐to‐lymphocyte ratio; PNI, prognostic nutritional index; SMI, skeletal muscle index; WBC, white blood cell.

^a^ Because the date interval of SMI measurement was different for each patient, it was converted into a change in 50 days.

^b^ PNI, prognostic nutritional index, calculated as (10 × Albumin [g/dL] + 0.005 × ALC).

### Survival outcome and prognostic factors

**Figure 1:**
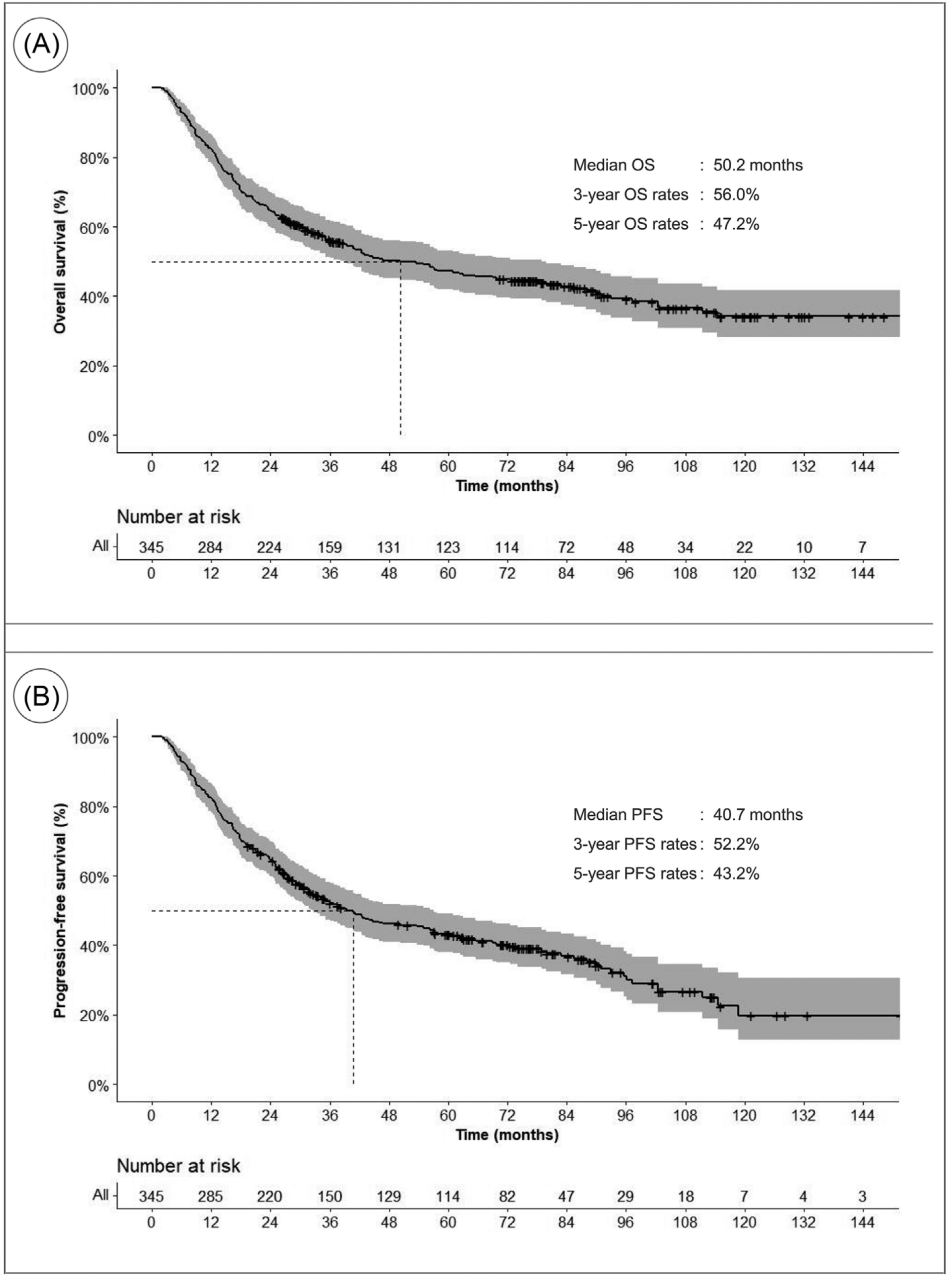
(A) Overall survival. (B) Progression‐free survival of patients with locally advanced oesophageal cancer receiving neoadjuvant chemoradiation therapy.

During a median follow‐up period of 32.8 months (range, 2.0–176.2), median OS for all patients was 50.2 months, and 1‐, 3‐ and 5‐year OS rates were 82.3%, 56.0%, and 47.2%, respectively (Figure 1A). The median PFS was 40.7 months, and the 1‐, 3‐, and 5‐year PFS rates were 82.3%, 52.2%, and 43.2%, respectively (Figure 1B).

**Figure 2:**
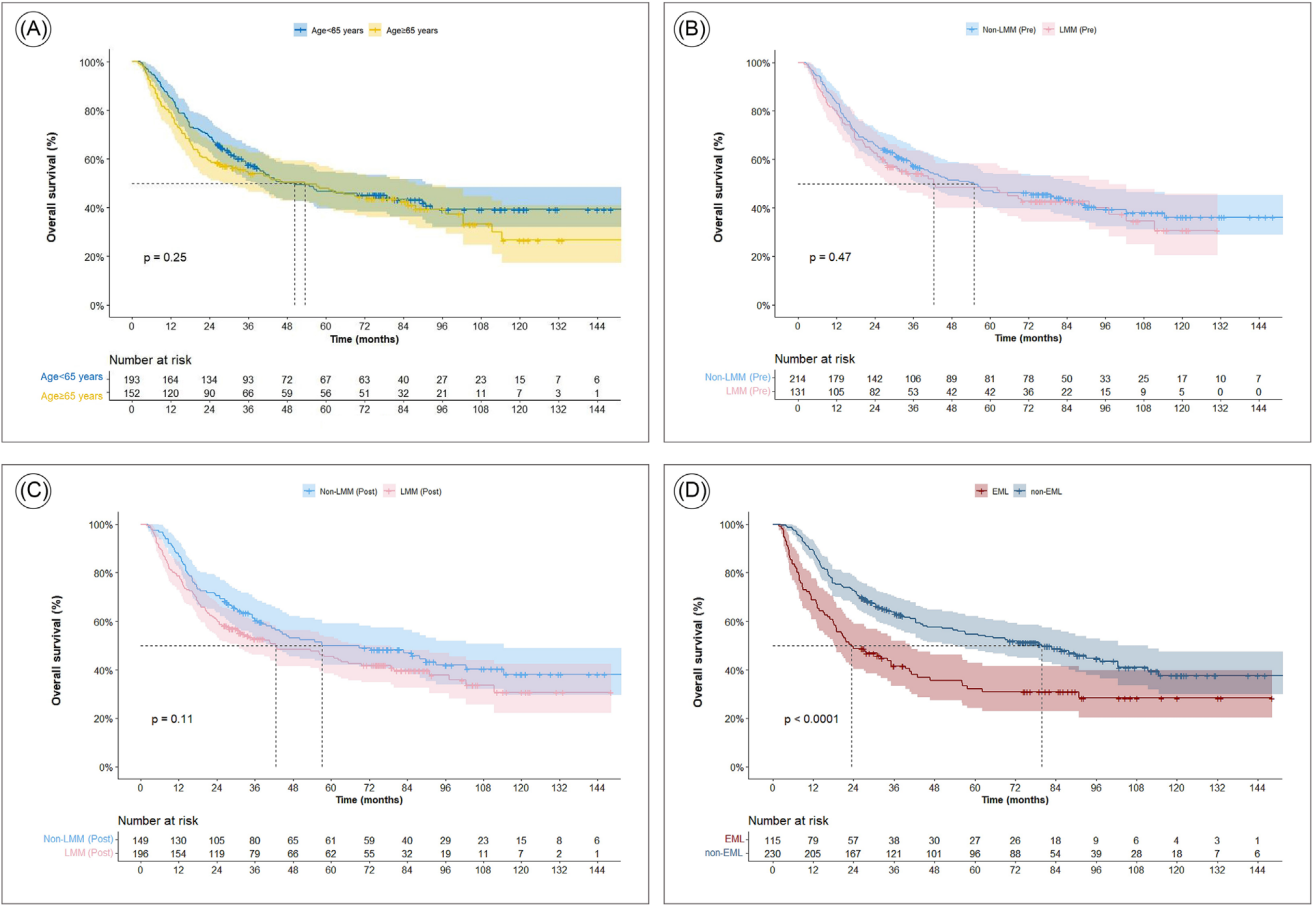
Overall survival according to (A) age (B) low muscle mass before neoadjuvant chemoradiation therapy (NACRT) (C) low muscle mass after NACRT (D) muscle loss during NACRT. EML, excessive muscle loss; non‐EML, nonexcessive muscle loss.

**Figure 3:**
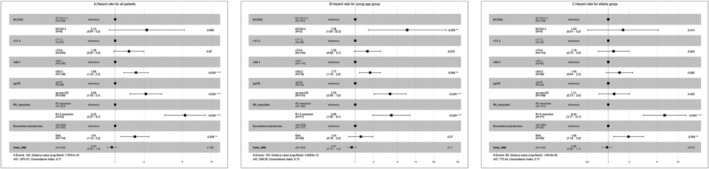
Multivariate analysis of factors affecting survival for (A) all patients, (B) patients aged <65 years, (C) patients aged ≥65 years. BMI, body mass index; ECOG, European Cooperative Oncology Group performance status; EML; excessive muscle loss; non‐EML, non‐excessive muscle loss; ypCR, pathologic complete response after neoadjuvant chemoradiation therapy.

Univariate analysis was conducted to determine the factors affecting OS. ECOG PS, clinical T and N stages, ypCR status, R0 resection status, ΔBMI, and EML (ΔSMI ≤−10%) were identified as significant factors (Table 2). However, age and low muscle mass before and after treatment did not appear to affect OS, and the log‐rank test showed similar results (3‐year OS rates: <65 years vs. ≥65 years, 57.7% vs. 54.0%, *P* = 0.247) (Figure 2A). Inflammatory and nutritional index values and their changes were not associated with survival. Multivariate analysis showed that OS was poor in patients with clinical stage N2–3 disease, non‐ypCR, R1–2 resection, and EML (Table 2, Figure 3A).

**Table 2:** Univariate and multivariate analyses of variables influencing overall survival after neoadjuvant chemoradiation therapy in patients with locally advanced oesophageal cancer

Variables	Overall survival				
	Univariate analysis		Multivariate analysis		Comparison group
	HR (95% CI)	*P* value	HR (95% CI)	*P* value	
Age	1.01 (0.99–1.03)	0.512			[Continuous]
ECOG PS, 2	2.57 (1.05–6.27)	0.038	2.13 (0.87–5.30)	0.099	0–1
Smoking, current smoker	0.91(0.68–1.20)	0.501			Never, Ex‐smoker
Clinical T stage, cT3–4	1.66 (1.17 2.36)	0.005	1.39 (0.97–2.00)	0.070	cT0–1
Clinical N stage, cN2–3	1.63 (1.31–2.03)	<0.001	1.65 (1.23–2.20)	<0.001	cN0–1
ypCR	2.52 (1.74–3.66)	<0.001	2.09 (1.43–3.10)	<0.001	non‐ypCR
Extent of resection, R1–2 resection	5.53 (3.45–8.87)	<0.001	5.33 (3.27–8.70)	<0.001	R0 resection
CTV	1.00 (1.00–1.00)	1.000			[Continuous]
Radiation therapy technique, IMRT	1.21 (0.90–1.64)	0.210			3D‐CRT
Low muscle mass before NACRT, yes	1.19 (0.87–1.64)	0.270			No
Low muscle mass after NACRT, yes	1.16 (0.75–1.79)	0.509			No
ΔBMI, kg/m^2^	0.84 (0.76–0.92)	<0.001	0.93 (0.83–1.00)	0.165	[Continuous]
ΔSMI ≤−10%	1.79 (1.34–2.39)	<0.001	1.61 (1.15–2.20)	0.006	> − 10%
ΔWBC, ×10^3^/μL	1.01 (0.97–1.05)	0.714			[Continuous]
ΔPlatelet count, ×10^3^/μL	1.00 (1.00–1.00)	0.229			[Continuous]
ΔANC, ×10^3^/μL	1.00 (0.96–1.05)	0.907			[Continuous]
ΔALC, ×10^3^/μL	1.03 (0.87–1.23)	0.712			[Continuous]
ΔAlbumin, g/dL	1.11 (0.82–1.50)	0.512			[Continuous]
ΔNLR	1.01 (1.00–1.02)	0.131			[Continuous]
ΔPLR	1.00 (1.00–1.00)	0.627			[Continuous]
ΔPNI	1.01 (0.99–1.03)	0.483			[Continuous]

*P* values <0.05 were considered statistically significant.

3D‐CRT, three‐dimensional conformal radiation therapy; ALC, absolute lymphocyte count; ANC, absolute neutrophil count; BMI, body mass index; CI, confidence interval; CR, complete response; CTV, clinical target volume; ECOG PS, Eastern Cooperative Oncology Group performance status; HR, hazard ratio; IMRT, intensity‐modulated radiation therapy; NACRT, neoadjuvant chemoradiotherapy; NLR, neutrophil‐to‐lymphocyte ratio; PLR, platelet‐to‐lymphocyte; SMI, skeletal muscle index; WBC, white blood cell.

### Impact of muscle mass by age groups

With elderly patients defined as those aged ≥65 years, 193 of patients (55.9%) belonged to the young age group and 152 patients (44.1%) belonged to the elderly group (Table 3). In the young and elderly groups, patients with low muscle mass before NACRT were 135 (69.9%) and 110 (72.4%) (*P* = 0.710), and 162 (83.9%) and 139 (91.4%) (*P* = 0.056) after NACRT, respectively. Fifty‐six patients (29.0%) and 59 patients (38.8%) had EML in each age group (*P* = 0.072).

**Table 3:** Patient characteristics by age group

	No. of patients (%)		*P* value
	Age <65 years (*n* = 193)	Age ≥65 years (*n* = 152)	
Age, median, years [IQR]	59 [55–61]	69 [67–73]	<0.001
ECOG PS			
ECOG PS 0–1	191 (99.0)	148 (97.4)	0.477
ECOG PS 2	2 (1.0)	4 (2.6)	
Smoking			
Never, ex‐smoker	65 (33.7)	97 (63.8)	<0.001
Current smoker	128 (66.3)	55 (36.2)	
Clinical T stage			
cT1–2	49 (25.4)	42 (27.6)	0.729
cT3–4	144 (74.6)	110 (72.4)	
Clinical N stage			
cN0–1	114 (59.1)	92 (60.5)	0.870
cN2–3	79 (40.9)	60 (39.5)	
Pathologic T stage			
pT0	91 (47.2)	73 (48.0%	0.958
pT+	102 (52.8)	79 (52.0)	
Pathologic N stage			
pN0	89 (46.1)	79 (52.0)	0.331
pN+	104 (53.9)	73 (48.0)	
Pathologic CR after NACRT			
ypCR	52 (26.9)	44 (28.9)	0.771
Non‐ypCR	141 (73.1)	108 (71.1)	
Extent of resection			
R0 resection	182 (94.3)	141 (92.8)	0.720
R1–2 resection	11 (5.7)	11 (7.2)	
Location of tumour			
Upper	51 (26.4)	34 (22.4)	0.312
Middle	72 (37.3)	69 (45.4)	
Lower	70 (36.3)	49 (32.2)	
CTV, median, cm^3^ [IQR]	215.1 [156.9–274.8]	211.5 [164.5–261.6]	0.834
Radiation therapy technique			
3D‐CRT	113 (58.5)	93 (61.2)	0.700
IMRT	80 (41.5)	59 (38.8)	
Low muscle mass before NACRT			
Yes	135 (69.9)	110 (72.4)	0.710
No	58 (30.1)	42 (27.6)	
Low muscle mass after NACRT			
Yes	162 (83.9)	139 (91.4)	0.056
No	31 (16.1)	13 (8.6)	
Excessive muscle loss			
Yes	56 (29.0)	59 (38.8)	0.072
No	137 (71.0)	93 (61.2)	

*P* values <0.05 were considered statistically significant.

3D‐CRT, three‐dimensional conformal radiation therapy; CR, complete response; CTV, clinical target volume; ECOG PS, European Cooperative Oncology Group performance status; IMRT, intensity‐modulated radiation therapy; IQR, interquartile range; NACRT, neoadjuvant chemoradiotherapy.

**Figure 4:**
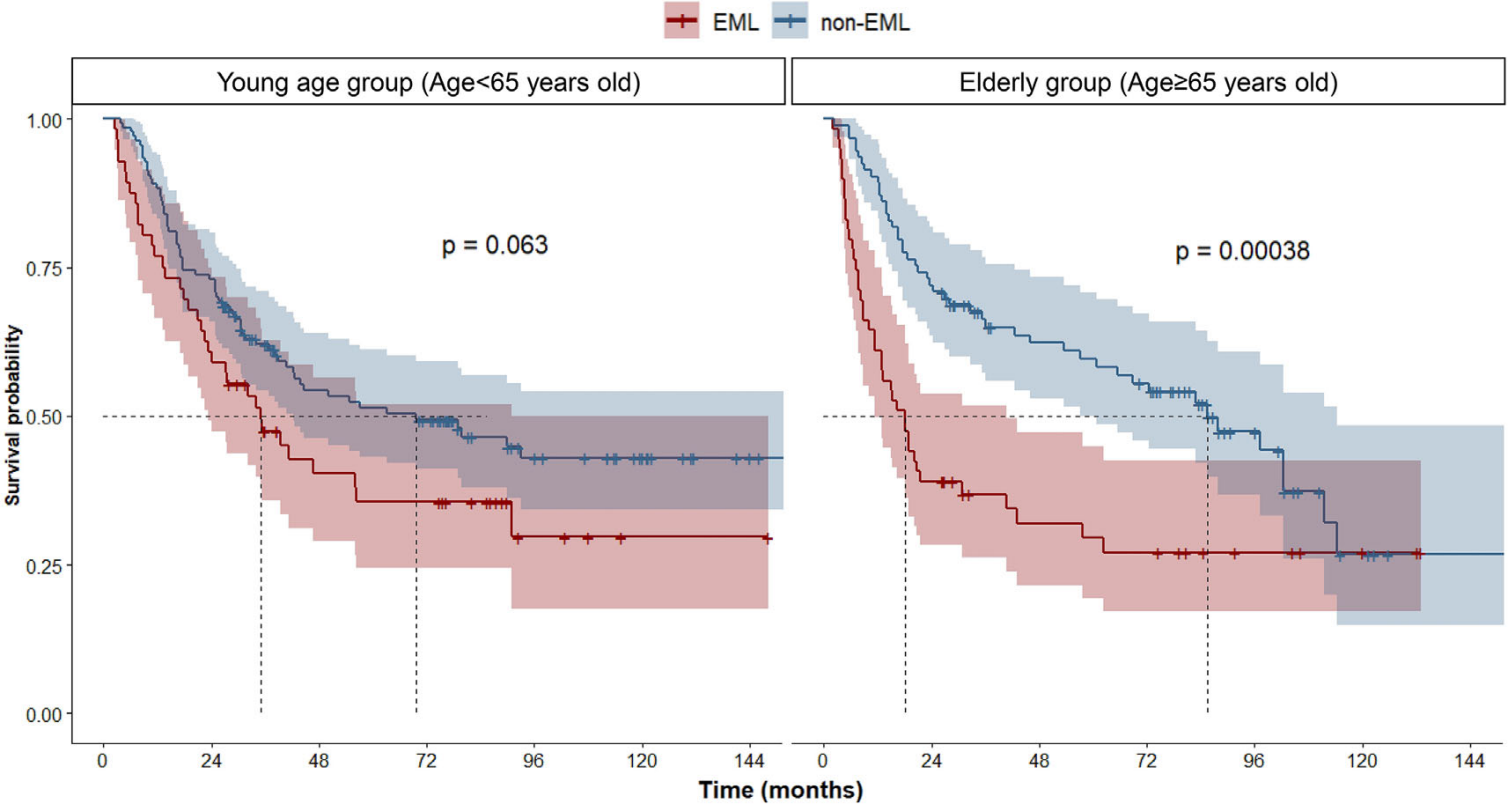
Survival probability according to muscle loss by age. EML, excessive muscle loss; non‐EML, nonexcessive muscle loss.

Patients in each age group were divided into EML and non‐EML groups, and the distribution of patient characteristics was even, except for tumour location in elderly patients (Table 4). The average and SD of ΔSMI in the EML and non‐EML groups were −14.1 ± 4.3 and −3.6 ± 4.3 in the young age group and −13.6 ± 3.3 and −3.5 ± 4.6 in the elderly group. In the younger age group, the 3‐year OS rates in the EML and non‐EML groups were 47.4% and 62.1%, respectively, showing only a trend toward worse OS in the EML group (*P* = 0.063). In contrast, in the elderly group, the 3‐year OS was 36.8% and 64.9%, respectively, and the EML group had significantly worse OS (*P* < 0.001) (Figure 4). Multivariate subgroup analysis was performed for each age group. In the young group, ECOG PS, clinical nodal stage, ypCR, and extent of resection were identified as factors influencing OS; however, only the extent of resection (*P* < 0.001) and excessive muscle loss (*P* = 0.008) were associated with poor OS in the elderly group. (Figure 3B,C). With ΔSMI of 0 set as the reference and the risk of death calculated with a Cox‐proportional model according to the change in ΔSMI, the hazard ratio for OS according to the degree of muscle loss in each age group is shown in *Figure* *S3*.

**Table 4:** Baseline characteristics of patients with excessive and non‐excessive muscle loss in each age group

	Number of patients (%)					
	Age <65 years (*n* = 193)			Age ≥65 years (*n* = 152)		
	EML (*n* = 56)	Non‐EM (*n* = 137)	*P* value	EML (*n* = 59)	Non‐EML (*n* = 93)	*P* value
Age, median, years [range]	59 [43–64]	59 [36–64]	0.505	70 [65–83]	69 [65–79]	0.094
ECOG PS						
ECOG PS 0–1	56 (100.0)	135 (98.5)	0.900	57 (96.6)	91 (97.8)	1.000
ECOG PS 2	0 (0.0)	2 (1.5)		2 (3.4)	2 (2.2)	
Clinical T stage						
cT1–2	11 (19.6)	38 (27.7)	0.322	15 (25.4)	27 (29.0)	0.765
cT3–4	45 (80.4)	99 (72.3)		44 (74.6)	66 (71.0)	
Clinical N stage						
cN0–1	29 (51.8)	85 (62.0)	0.248	33 (55.9)	59 (63.4)	0.452
cN2–3	27 (48.2)	52 (38.0)		26 (44.1)	34 (36.6)	
Complete response						
ypCR	12 (21.4)	40 (29.2)	0.355	16 (27.1)	28 (30.1)	0.832
Non‐ypCR	44 (78.6)	97 (70.8)		43 (72.9)	65 (69.9)	
Extent of resection						
R0	54 (96.4)	128 (93.4)	0.636	53 (89.8)	88 (94.6)	0.429
R1–2	2 (3.6)	9 (6.6)		6 (10.2)	5 (5.4)	
Location of tumour						
Upper	13 (23.2)	38 (27.7)	0.081	10 (16.9)	24 (25.8)	0.042
Middle	16 (28.6)	56 (40.9)		23 (39.0)	46 (49.5)	
Lower	27 (48.2)	43 (31.4)		26 (44.1)	23 (24.7)	
RT technique						
3D‐CRT	29 (51.8)	84 (61.3)	0.290	33 (55.9)	60 (64.5)	0.375
IMRT	27 (48.2)	53 (38.7)		26 (44.1)	33 (35.5)	
Low muscle mass, before CCRT						
Yes	39 (69.6)	96 (70.1)	1.000	42 (71.2)	68 (73.1)	0.941
No	17 (30.4)	41 (29.9)		17 (28.8)	25 (26.9)	
Low muscle mass, after CCRT							
Yes	52 (92.9)	110 (80.3)	0.052	57 (96.6)	82 (88.2)	0.130
No	4 (7.1)	27 (19.7)		2 (3.4)	11 (11.8)	

*P* values <0.05 were considered statistically significant. Since the date interval of SMI measurement was different for each patient, it was converted into a change in 50 days.

3D‐CRT, three‐dimensional conformal radiation therapy; CR, complete response; ECOG PS, European Cooperative Oncology Group performance status; EML, excessive muscle loss; IMRT, intensity‐modulated radiation therapy; RT, radiation therapy.

## Discussion

In this study, elderly patients showed a comparable OS when NACRT followed by surgery was used to treat LAEC. In addition, EML after NACRT was strongly associated with poor OS, especially in elderly patients. These results indicate that elderly patients may be candidates for NACRT followed by surgery. However, they are more susceptible to the adverse impacts of muscle loss on survival, and there may be room for improvement in survival through interventions to reduce muscle loss.

Although NACRT followed by surgery is recommended for LAEC, treating elderly patients in clinical practice is challenging. The inclusion criteria of the CROSS were limited to the age ≤75 years and in the NEOCRTEC trial, it was ≤70. The median age of patients participating in the CROSS trial was 60 years (range, 36–79), and in the NEOCRTEC study, the mean age was 56 years (range, 31–70), with only 59 patients (26.3%) aged ≥60 years in the NACRT arm.^4^, ^20^ The median age of the patients in this study was 63 years, and 44.1% were aged >65 years. Thus, more elderly patients are included than in a randomized controlled trial, so there are practical difficulties in treating and managing them in the real world.^21^

Table S2 summarizes the survival of elderly patients with oesophageal cancer.^7^, ^8^, ^9^, ^10^ Elderly patients were defined using various age cut‐offs, ranging from 65 to 75 years, and most studies included heterogeneous treatment groups of patients who underwent either definitive or neoadjuvant RT. In a study by Miyata et al., which included only those who underwent neoadjuvant therapy, the 5‐year survival rates of patients <70 years, 70 to 75 years, 75–80 years, and ≥80 years were 52.4%, 50.2%, 38.1%, and 29.3%, respectively, and it was significantly worse in the 75–80 and ≥80 age groups.^8^ In our study, 29 and three patients aged 75–80 and ≥80 years and the 5‐year survival rates were 42.0% and 0% (*Table* *S3*, *Figure* *S4A*, *B*), respectively. The survival rate tends to decrease in the oldest patients aged over 80 years, and similar results were reported in a study by Rahimi et al. (median OS; 75–80 years vs. ≥80 years, 44 months vs. 23 months, *P* = 0.17).^9^ Despite these trends, we observed comparable survival outcomes between the young (age ≤65 years old) and elderly (age >65 years old) groups in our study. Considering that the majority of elderly patients receiving active cancer treatment in the real world fall within the age range of their 60s and 70s, we believe that this study supports the application of NACRT in patients with advanced age. However, it is crucial to acknowledge that there may be a potential bias in the selection of patients for this treatment, as it tends to favour individuals who are relatively medically fit. Consequently, caution should be exercised when attempting to generalize these findings beyond this specific patient cohort.

The importance of low muscle mass has already been proven, and patients with sufficient muscle mass show rapid recovery and prolonged survival.^22^, ^23^, ^24^ Studies on the prognostic effect of low muscle mass on the survival of patients with NAT are presented in *Table* *S4*. A previous study in out center included 248 patients of all ages, and the 5‐year survival rates of the EML and non‐EML groups were 45.1% and 69.8%, respectively (*P* < 0.001).^16^ However, there was no difference in survival according to age, consistent with the results of this study. There is some controversy regarding the impact of muscle mass on patient survival. In the study by Haung et al., the median OS of patients with sarcopenia was remarkably shorter (median OS; sarcopenia vs. non‐sarcopenia, 21.9 months vs. 30.5 months, *P* < 0.001). In contrast, in a study by Panje et al., there was no significant difference in OS (*P* = 0.72) but there were more patients with grade 3 toxicity during NACRT in the sarcopenia group (sarcopenia vs. non‐sarcopenia, 83.3% vs. 52.4%, *P* = 0.041).^25^, ^26^ Furthermore, there have been several studies in Japan regarding the impact of sarcopenia on survival outcomes, with neoadjuvant chemotherapy being the primary treatment modality.^27^, ^28^ In particular, Onishi et al. have demonstrated that not only sarcopenia alone, but also the co‐existence of obesity in elderly patients, exacerbates the deterioration of survival.^29^ In our study, the amount of muscle mass loss during treatment was more important than whether patients had loss muscle mass before or after radiation treatment, which was true not only in the findings of Järvinen et al. but also in studies of other types of cancer.^30^, ^31^, ^32^, ^33^ Although chemotherapy alone as a neoadjuvant treatment, similar findings have been observed in the elderly population in Japan, wherein the loss of muscle during treatment was found to be more closely associated with survival outcomes than sarcopenia itself.^34^ Therefore, many physicians and patients should be aware that the maintenance of muscle mass and nutritional supplementation during treatment are highly related to survival, and considering the differences in influence according to age, elderly patients need more careful management. Muscle loss can result from inadequate food intake due to anorexia or dysphagia, reduced nutrient absorption due to mucosal disruption, decreased physical activity, and cancer‐related immune activation due to hormone‐induced metabolic changes and cytokine release.^35^, ^36^ While the exact mechanism linking skeletal muscle loss to decreased survival remains unclear, hypotheses have suggested potential associations with inflammation, reduced immune activity, and inactivity.^33^ In this study, we also explored the possible correlation between inflammatory indices and muscle loss, but no significant associations were observed (*Table* *S5*). Therefore, further pre‐clinical and clinical research is warranted to gain a deeper understanding of these mechanisms. Nonetheless, at the current state of knowledge, it is advisable to consider active intervention through continuous monitoring and evaluation of nutritional status, body composition, and complications should be performed during the course of treatment to reduce complications and maximizing quality of life and treatment efficacy.^36^ Moreover, in the management of cancer treatment in the elderly, not only does nutrition determine morbidity, mortality, and quality of life but cognitive and functional status, social and family support, drug use, and mood should also be managed; therefore, it would be desirable to have comprehensive support for the geriatric sector.^37^, ^38^

The strengths of this study are that there was a younger control group unlike a single‐arm study with the elderly alone and that the body indices of a large number of patients could be measured. Moreover, by comparing the differences before and after NACRT, it was possible to provide evidence for the importance of providing support to patients during treatment. The limitations of this study are that it was retrospective in nature; no toxicity data were investigated, and only males and limited treatment groups were included. A study of simple associations is also a limitation, and it would be beneficial to conduct further investigations using an in‐house nutritional support protocol to determine whether active interventions can improve survival.

## Conclusions

NACRT is effective and applicable to LAEC patients with advanced‐age LAECs. EML may be strongly associated with a deteriorated OS in elderly patients undergoing NACRT followed by surgery for LAEC. Continuous monitoring and nutritional and physical support are necessary during treatment. Therefore, strategies for decreasing muscle loss in these patients should be investigated.

## Conflict of interest

The authors declare that they have no conflicts of interest.

## Supporting information


**Figure S1.** Flow diagram.
**Figure S2.** An example of skeletal muscle index (SMI) delineation (A) a 69‐year‐old male patient before neoadjuvant concurrent chemoradiotherapy (CCRT) with an SMI of 49.7 cm2/m2, and (B) the same patient after CCRT with an SMI of 36.6 cm2/m2.
**Figure S3.** Mortality hazard ratio as a function of muscle loss by age.
**Figure S4.** Overall survival in patients with oesophageal cancers stratified by age at the time of neoadjuvant chemoradiation therapy: (A) age 75 (B) age 80.

**Table S1.** Patient characteristics (*n* = 345).
**Table S2.** Studies on survival of elderly patients with oesophageal cancer.
**Table S3.** Oncological outcomes in each age group.
**Table S4.** Prognostic impact of muscle mass on the survival of patients with oesophageal cancer who underwent neoadjuvant therapy.
**Table S5.** The correlation between changes in nutritional indices and changes in skeletal muscle index before and after neoadjuvant therapy.

## Data Availability

Data supporting the findings of this study are available upon request from the corresponding authors.
