# Supplementary material for: Prognostic impact of muscle mass loss in elderly patients with oesophageal cancer receiving neoadjuvant chemoradiation therapy
Source: J Cachexia Sarcopenia Muscle. 2024 Apr 13;15(3):1167–76. doi: 10.1002/jcsm.13462 (PMC11154764; doi:10.1002/jcsm.13462)

**Figure S1** Flow diagram


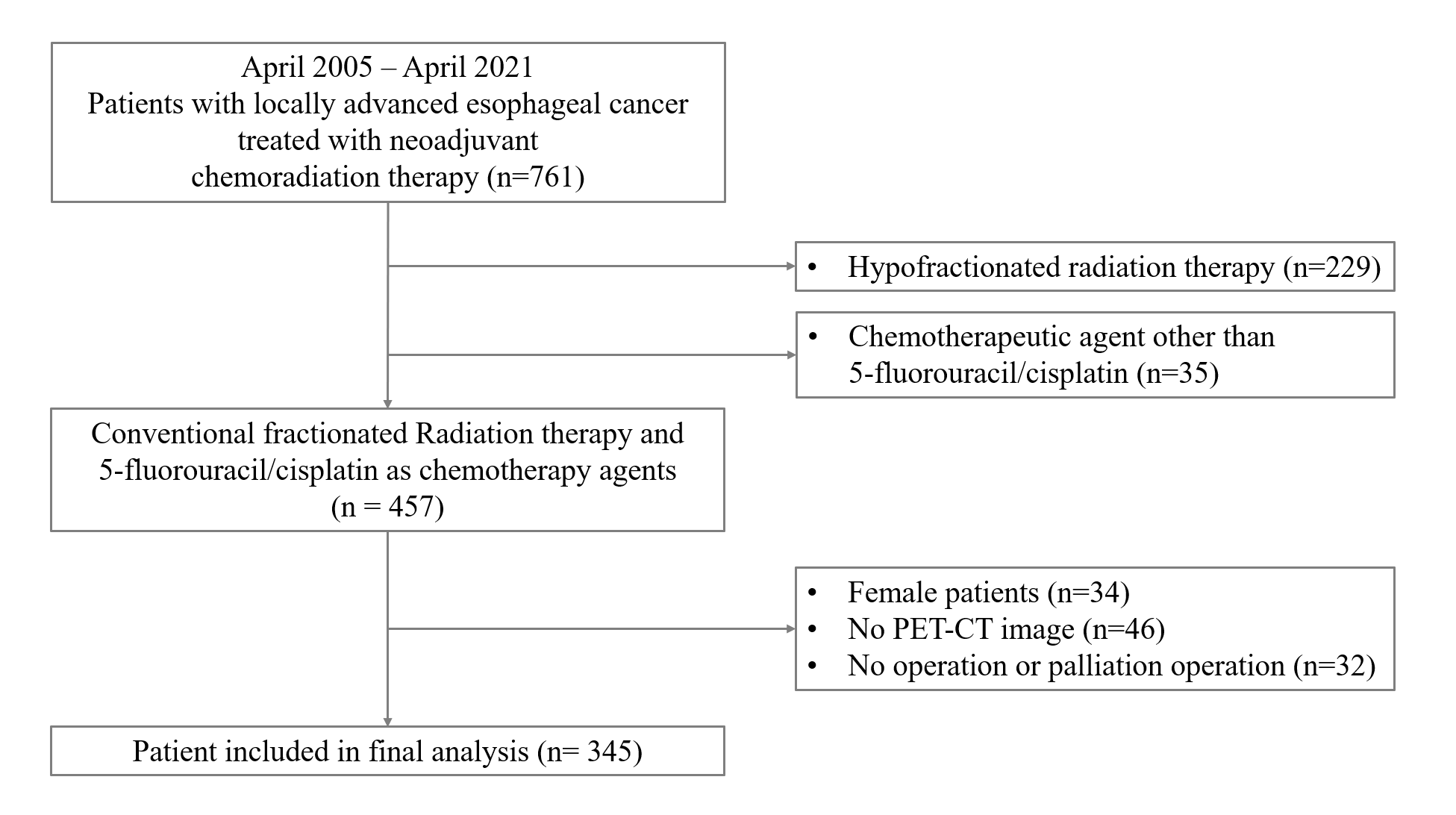


**Figure S2** An example of skeletal muscle index (SMI) delineation (A) a 69-year-old male patient before neoadjuvant concurrent chemoradiotherapy (CCRT) with an SMI of 49.7 cm2/m2, and (B) the same patient after CCRT with an SMI of 36.6 cm2/m2


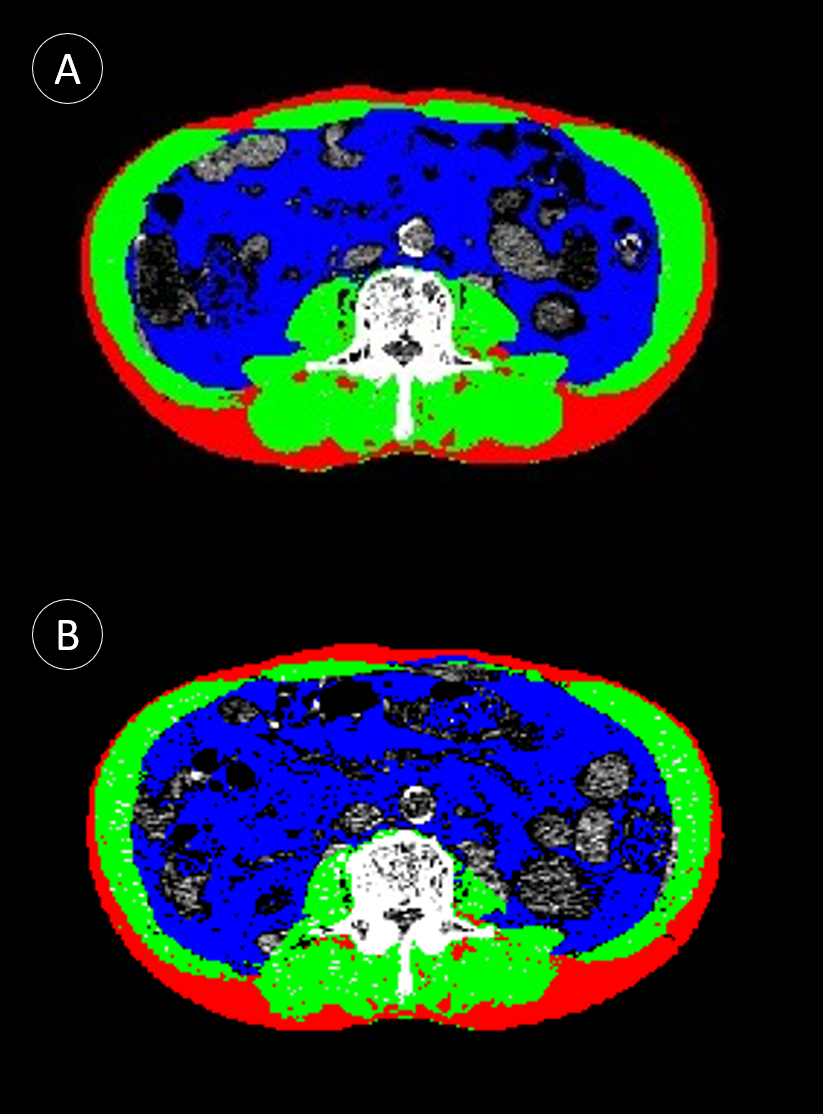


**Figure S3** Mortality hazard ratio as a function of muscle loss by age.


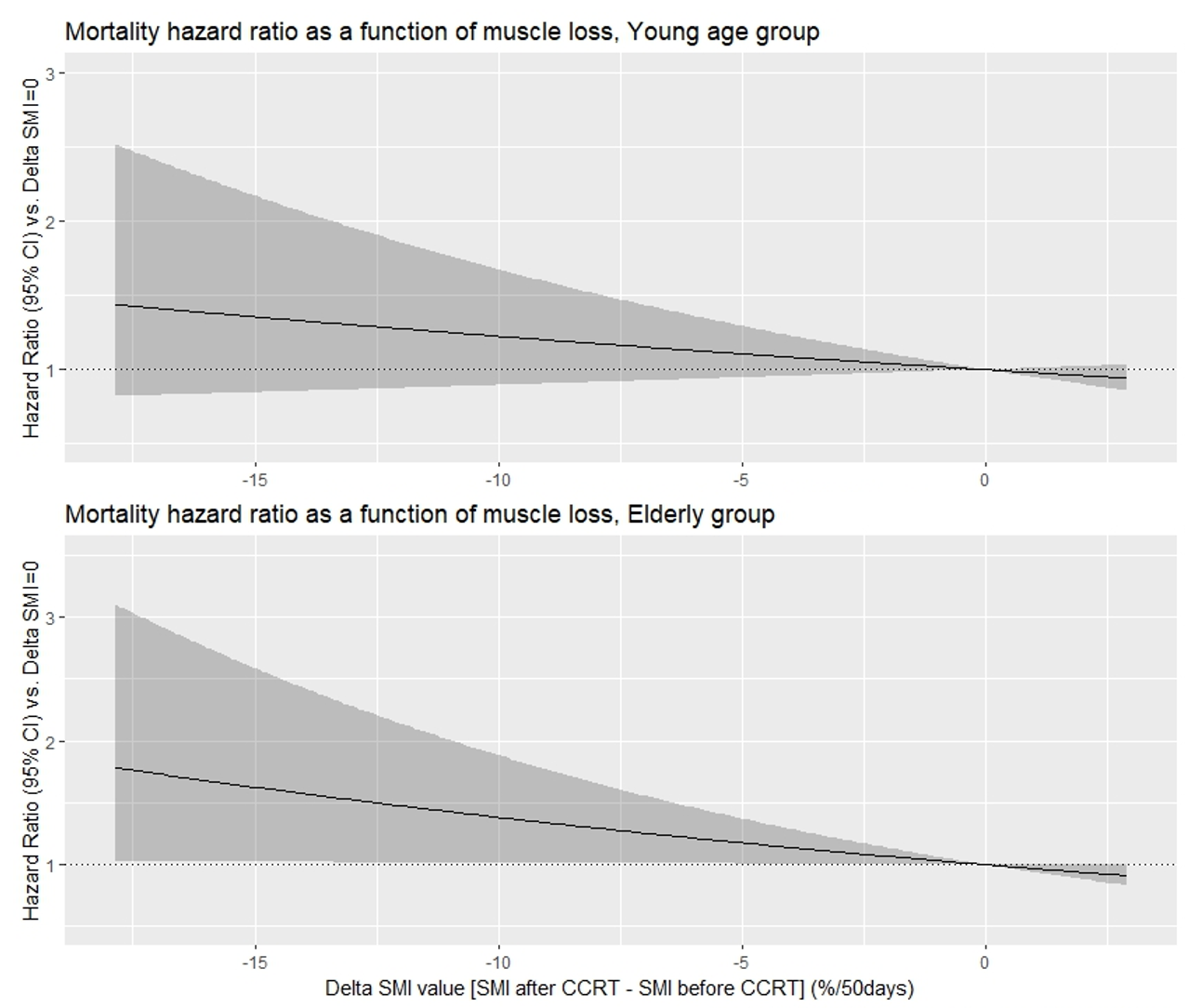


CI, confidence interval; SMI, skeletal muscle index; CCRT, concurrent chemoradiation therapy.

**Figure S4** Overall survival in patients with esophageal cancers stratified by age at the time of neoadjuvant chemoradiation therapy: (A) age 75 (B) age 80.


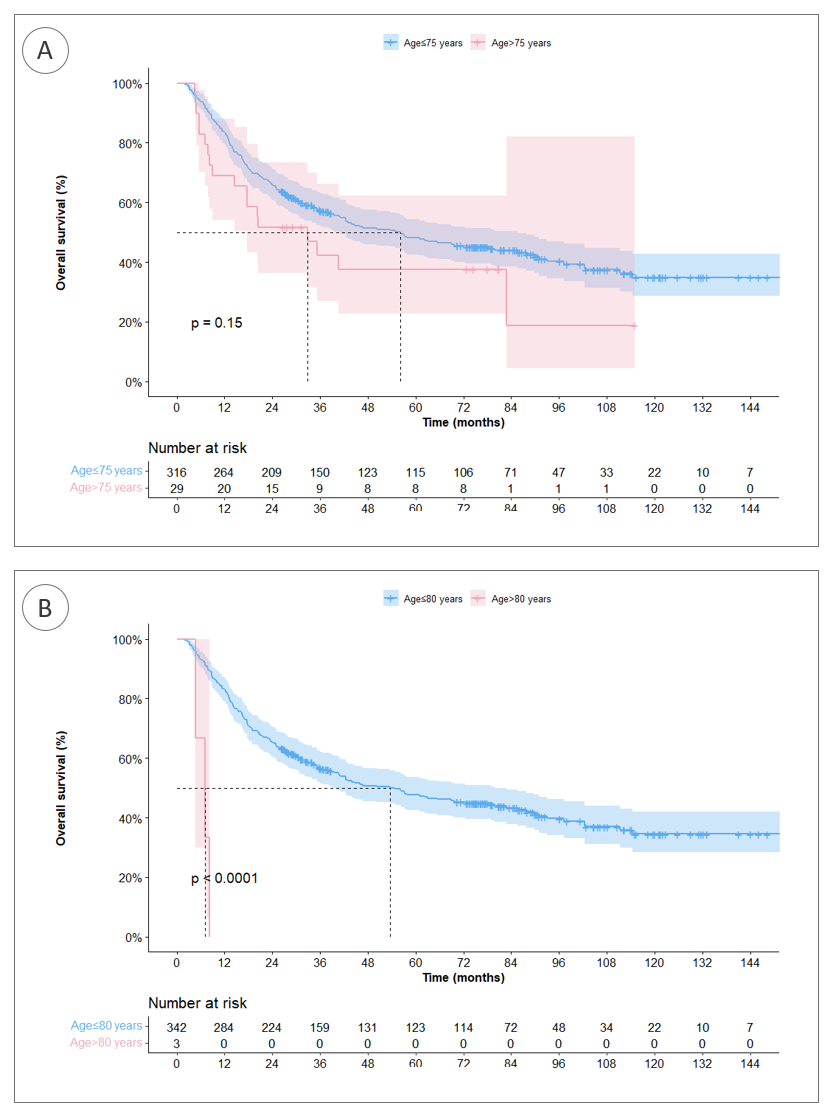

Supplement: Supplementary file 1 — Figure S1. Flow diagram. Figure S2. An example of skeletal muscle index (SMI) delineation (A) a 69‐year‐old male patient before neoadjuvant concurrent chemoradiotherapy (CCRT) with an SMI of 49.7 cm2/m2, and (B) the same patient after CCRT with an SMI of 36.6 cm2/m2. Figure S3. Mortality hazard ratio as a function of muscle loss by age. Figure S4. Overall survival in patients with oesophageal cancers stratified by age at the time of neoadjuvant chemoradiation therapy: (A) age 75 (B) age 80. [file JCSM-15-1167-s002.docx]
