# Supplementary material for: Prognostic impact of muscle mass loss in elderly patients with oesophageal cancer receiving neoadjuvant chemoradiation therapy
Source: J Cachexia Sarcopenia Muscle. 2024 Apr 13;15(3):1167–76. doi: 10.1002/jcsm.13462 (PMC11154764; doi:10.1002/jcsm.13462)
Supplement: Supplementary file 2 — Table S1. Patient characteristics (n = 345). Table S2. Studies on survival of elderly patients with oesophageal cancer. Table S3. Oncological outcomes in each age group. Table S4. Prognostic impact of muscle mass on the survival of patients with oesophageal cancer who underwent neoadjuvant therapy. Table S5. The correlation between changes in nutritional indices and changes in skeletal muscle index before and after neoadjuvant therapy. [file JCSM-15-1167-s001.docx]

**Table S1** Patient characteristics (n=345)

|  | No. of patients (%) |
| --- | --- |
| Age, median, years [range] | 63 [36–83] |
| ECOG PS |  |
| ECOG PS 0–1 | 339 (98.3) |
| ECOG PS 2 | 6 (1.7) |
| Smoking |  |
| Never, Ex-smoker | 162 (47.0) |
| Current smoker | 183 (53.0) |
| Clinical T stage |  |
| cT1–2 | 91 (26.4) |
| cT3–4 | 254 (73.6) |
| Clinical N stage |  |
| cN0–1 | 206 (59.7) |
| cN2–3 | 139 (40.3) |
| Pathologic T stage |  |
| pT0 | 164 (47.5) |
| pT+ | 181 (52.5) |
| Pathologic N stage |  |
| pN0 | 168 (48.7) |
| pN+ | 177 (51.3) |
| Pathologic complete response after NACRT |  |
| ypCR | 96 (27.8) |
| non-ypCR | 249 (72.2) |
| Extent of resection |  |
| R0 resection | 323 (93.6) |
| R1-2 resection | 22 (6.4) |
| Location of tumor |  |
| Upper | 85 (24.6) |
| Middle | 141 (40.9) |
| Lower | 119 (34.5) |
| CTV, median, cm^3^ [range] | 228.5 [35.1–929.2] |
| Radiation therapy technique |  |
| 3D-CRT | 206 (59.7) |
| IMRT | 139 (40.3) |
| **Low muscle mass** before NACRT |  |
| Yes | 245 (71.0) |
| No | 100 (29.0) |
| **Low muscle mass** after NACRT |  |
| Yes | 301 (87.2) |
| No | 44 (12.8) |

ECOG PS, European Cooperative Oncology Group performance status; NACRT, neoadjuvant chemoradiotherapy; CR, complete response; CTV, clinical target volume; 3D-CRT, three-dimensional conformal radiation therapy; IMRT, intensity-modulated radiation therapy

**Table S2** Studies on survival of elderly patients with esophageal cancer

|  | OURS | Linde et al. | Rahimy et al. | Walter et al. | Miyata et al. |
| --- | --- | --- | --- | --- | --- |
| Year of publication | 2023 | 2022 | 2020 | 2018 | 2015 |
| No. of patients | 345 | 67 | 89 | 55 | 722 |
| Median age (range) | 63 (36–83) | 71 (65–82) | 80 (76–84) | 75 (70–85) | Unknown |
| Definition of elderly (years) | 65 | 65 | 75 | 70 | 70 |
| TNM stage | T1 24, T2 67, T3 247, T4 7  N0 16, N+ 329 | II 4, III 48, IV 7  Unknown 8 | I 4, II 16, III 55, IV 14s | T1 1, T2 11, T3 32, T4 11  N0 23, N+ 32 | I 166/ II 154  III 301 / IV 101 |
| Aim of radiation therapy | NAT | NAT, Definitive | NAT, Definitive, PORT | NAT, Definitive | NAT |
| Patients with NACRT | 100.0% | 34.3% | 23.6% | 14.6% | 14.9% |
| Total radiation dose | 44 Gy | DCRT: 50.4 Gy+9Gy  NACRT: 41.4 Gy | 50 Gy  (range, 45–66) | 59.4 Gy  (range, 10.8-66.0) | 40–60 Gy |
| RT technique | 3D-CRT/IMRT | 2D/3D-CRT/IMRT | 3D-CRT/IMRT | 3D-CRT/IMRT | Unknown |
| Patients with CCRT | 100.0% | 100.0% | 91.0% | 85.5% | 14.9% |
| Chemotherapy regimen | FP | TC, FP | TC, FP | FP, mitomycin C/5-FU | ACF, DCF |
| Follow-up period (months) | 32.8 (range, 2.0–176.2) | 24.0 | 16.0 (IQR; 6.0–29.0) | 11.0 (1.0–68.0) | Unknown |
| Median OS (months) | 50.2 | 30.0 (All patients)  43.0 (NAT) | 28.0 | 12.0 | 5yr OS rates (%) by age  52.4/ 50.2/ 38.1 /29.3  (<70/70-75/75-80/>80yrs) |
| Factor associated with OS  (Univariate analysis) | ECOG, Clinical stage  ypCR, extent of resection  ΔBMI, ΔSMI | ECOG, Aim of radiation therapy | Clinical stage (IVB) | T stage, Comorbidity  Treatment period, Use of PET-CT for staging | Age, BMI, pathological stage, Postoperative complications |
| Difference in OS  between age groups | No difference  Median OS (*P* = 0.12)  <70 yrs, 57.2mo. vs.  ≥70 yr, 32.8 mo. | No difference  Median OS  (*P* = 0.632)  60-70 yr, 32 mo. vs.  ≥70 yr, 25 mo. | No difference  Median OS  (*P* = 0.17)  75-80 yr, 44 mo. vs.  ≥80 yr, 23 mo. | No difference  1yr OS rates  (*P* = 0.523)  <75 yrs, 54%  ≥75 yr, 63% | Poorer with age ≥75 yr  5yr OS rates (%)  75-80 yr, 38.1 (*P* = 0.011)  ≥80 yr, 29.3 (*P* = 0.002) |

NAT, neoadjuvant therapy; PORT, postoperative radiation therapy; RT, radiation therapy; 3D-CRT, 3-dimensional conformal radiation therapy; IMRT, intensity-modulated radiation therapy; CCRT, concurrent chemoradiation therapy; FP, 5-fluorouracil/cisplatin; TC, carboplatin/paclitaxel; ACF, 5-fluorouracil/cisplatin/adriamycin; DCF, 5-fluorouracil/cisplatin/docetaxel; IQR, interquartile range; OS, overall survival; ECOG, Eastern Cooperative Oncology Group performance status; ypCR, complete response after neoadjuvant radiation therapy and surgery; BMI, body mass index; SMI, skeletal muscle index; PET-CT, positron emission tomography-computed tomography

**Table S3** Oncological outcomes in each age group

| Age | Number of patients | | Progression-free survival | | | |
| --- | --- | --- | --- | --- | --- | --- |
|  | Total | Event | Median (months) | 1yr rate (%) | 2yr rate (%) | *P*-value |
| <65 yrs | 193 | 93 | 54.9 | 89.2 | 74.3 | 0.510 |
| ≥65 yrs | 152 | 60 | 95.6 | 88.4 | 71.0 |  |
| <70 yrs | 274 | 128 | 61.0 | 88.5 | 73.3 | 0.610 |
| ≥70 yrs | 71 | 25 | 85.5 | 90.3 | 70.7 |  |
| <75 yrs | 316 | 142 | 74.0 | 88.9 | 72.8 | 0.700 |
| ≥75 yrs | 29 | 11 | 35.2 | 87.4 | 73.5 |  |
| <80 yrs | 342 | 152 | 68.9 | 83.0 | 65.2 | 0.003 |
| ≥80 yrs | 3 | 1 | 8.3 | - | - |  |
| Age | Number of patients | | Overall survival | | | |
|  | Total | Event | Median (months) | 1yr rate (%) | 2yr rate (%) | *P*-value |
| <65 yrs | 193 | 103 | 50.2 | 85.0 | 68.9 | 0.250 |
| ≥65 yrs | 152 | 89 | 53.5 | 78.9 | 59.2 |  |
| <70 yrs | 274 | 148 | 57.2 | 83.9 | 67.5 | 0.120 |
| ≥70 yrs | 71 | 44 | 32.8 | 76.1 | 53.5 |  |
| <75 yrs | 316 | 174 | 56.2 | 83.5 | 65.8 | 0.150 |
| ≥75 yrs | 29 | 18 | 32.8 | 69.0 | 51.7 |  |
| <80 yrs | 342 | 189 | 53.5 | 83.0 | 65.2 | <0.001 |
| ≥80 yrs | 3 | 3 | 7.2 | 0.0 | 0.0 |  |

**Table S4** Prognostic impact of **muscle mass** on the survival of patients with esophageal cancer who underwent neoadjuvant therapy

|  | OURS | Yoon et al. | Huang et al. | Panje et al. | Järvinen et al. |
| --- | --- | --- | --- | --- | --- |
| Year of publication | 2023 | 2020 | 2020 | 2019 | 2018 |
| No. of patients | 345 | 248 | 107 | 61 | 115 |
| Median age (range) | 63 (36–83) | Mean 63.46  (SD; 7.63) | Mean 54.1  (SD; 7.5) | 61 (38–75) | Mean 63 (SD; 9) |
| Patients with **low muscle mass**^*^ | 245 (71.0%) | 156 (62.9%) | 65 (60.7%) | 29 (47.5%) | 92 (80.0%) |
| Total radiation dose | 44 Gy | 44 Gy (range, 40 – 50) | Unknown | 45 Gy | 45 Gy |
| Patients with CCRT | 100.0% | 100.0% | 100.0% | 100.0% | 75.7% |
| Chemotherapy regimen | FP | FP | FP | DC ± Cetuximab | FP |
| Median PFS (months) | 68.9 **(LMM: 68.9/**  **Non-LMM: 50.2)** | 5yr rate, 47.4% | S: 12.6, NS: 23.5 | Unknown | 2yr rate: S 54%, NS 44% |
| Median OS (months) | 50.2 **(LMM: 56.2/ Non-LMM: 46.7)** | 5yr rate, 62.5% | S: 21.9, NS: 30.5 | 5yr rate: S 47%, NS 57% | 2yr rate: S 60%, NS 61% |
| Factor associated with OS  (Univariate analysis) | ECOG, Clinical stage  ypCR, extent of resection  ΔBMI, ΔSMI | Clinical/ Pathologic stage, extent of resection, baseline albumin | Clinical stage,  Sarcopenia | (-) | ΔSMI |
| Difference in OS  by **muscle mass** | No difference  **Pre-LMM**, HR 1.19  (*P* = 0.270)  **Post-LMM**, HR 1.16  (*P* = 0.509) | No difference  Pre-S, HR 0.689  (*P* = 0.689)  Post-S, HR 0.877  (*P* = 0.669) | Significant difference  Pre-S, HR 2.45  (*P* < 0.001) | No difference  5yr OS rate (*P* = 0.72)  S:47%, NS: 57% | No difference  2yr OS rate (*P* = 0.74)  S: 60%, NS: 61% |
| Difference in OS  by muscle loss | Significant difference  ΔSMI< -10 (%/50days) | Significant difference  ΔSMI< -10 (%/50days) | Not evaluated | Not evaluated | Significant difference  ΔSMI< -2.98% (*P* = 0.022) |

CCRT, concurrent chemoradiation therapy; FP, 5-fluorouracil/cisplatin; DC, cisplatin/docetaxel; PFS, progression-free survival; **LMM, Low muscle mass;** S, patients with sarcopenia; NS, patients without sarcopenia; OS, overall survival; ECOG, Eastern Cooperative Oncology Group performance status; ypCR, complete response after neoadjuvant radiation therapy and surgery; BMI, body mass index; SMI, skeletal muscle index; **Pre-LMM, Low muscle mass before neoadjuvant therapy; Post-LMM, Low muscle mass after neoadjuvant therapy**

**^*^ Low muscle mass before neoadjuvant therapy**

**Table S5 The correlation between changes in nutritional indices and changes in skeletal muscle index before and after neoadjuvant therapy**

| **Variable** | **Correlation estimate** | ***P*-value** |
| --- | --- | --- |
| **ΔNLR vs. ΔSMI** | **-0.0922** | **0.0873** |
| **ΔPNR vs. ΔSMI** | **-0.0142** | **0.0081** |
| **ΔPNI^*^ vs. ΔSMI** | **0.2399** | **6.6026e-06** |

**NLR, neutrophil-to-lymphocyte ratio; SMI, Skeletal muscle index; PLR, platelet-to-lymphocyte ratio; PNI, prognostic nutritional index**

**^*^PNI, Prognostic Nutritional Index, calculated as (10 × Albumin [g/dL] + 0.005 × ALC)**
